# *RAD52* Functions in Homologous Recombination and Its Importance on Genomic Integrity Maintenance and Cancer Therapy

**DOI:** 10.3390/cancers11111622

**Published:** 2019-10-23

**Authors:** Augusto Nogueira, Mara Fernandes, Raquel Catarino, Rui Medeiros

**Affiliations:** 1 Molecular Oncology and Viral Pathology Group, IPO-Porto Research Center (CI-IPOP), Portuguese Institute of Oncology of Porto, 4200-072 Porto, Portugal; augusto.andre.nogueira@ipoporto.min-saude.pt (A.N.); mara.aires.fernandes@ipoporto.min-saude.pt (M.F.); raquelcatarino@gmail.com (R.C.); 2 Faculty of Medicine of University of Porto (FMUP), 4200-319 Porto, Portugal; 3 Biomedical Research Center (CEBIMED), Faculty of Health Sciences of Fernando Pessoa University, 4249-004 Porto, Portugal; 4 Research Department, Portuguese League against Cancer (NRNorte), 4200-172 Porto, Portugal

**Keywords:** DNA damage, DNA damage response, homologous recombination, *RAD52*, carcinogenesis, cancer therapy

## Abstract

Genomes are continually subjected to DNA damage whether they are induced from intrinsic physiological processes or extrinsic agents. Double-stranded breaks (DSBs) are the most injurious type of DNA damage, being induced by ionizing radiation (IR) and cytotoxic agents used in cancer treatment. The failure to repair DSBs can result in aberrant chromosomal abnormalities which lead to cancer development. An intricate network of DNA damage signaling pathways is usually activated to eliminate these damages and to restore genomic stability. These signaling pathways include the activation of cell cycle checkpoints, DNA repair mechanisms, and apoptosis induction, also known as DNA damage response (DDR)-mechanisms. Remarkably, the homologous recombination (HR) is the major DSBs repairing pathway, in which *RAD52* gene has a crucial repairing role by promoting the annealing of complementary single-stranded DNA and by stimulating *RAD51* recombinase activity. Evidence suggests that variations in *RAD52* expression can influence HR activity and, subsequently, influence the predisposition and treatment efficacy of cancer. In this review, we present several reports in which the down or upregulation of *RAD52* seems to be associated with different carcinogenic processes. In addition, we discuss *RAD52* inhibition in DDR-defective cancers as a possible target to improve cancer therapy efficacy.

## 1. Introduction

The human genome is constantly exposed to various genotoxic agents that have the potential to damage DNA. Causes of DNA-damaging insults include errors in DNA replication, telomeric shortening, and reactive oxygen species (ROS) which, in turn, are induced by endogenous and exogenous factors. Endogenous sources include metabolism and inflammation processes, while exposure to ionizing radiation (IR), UV light, individual diet, therapeutic agents, environmental, and pollutant chemical factors are exogenous sources [1].

Genomic stability maintenance and accurate transmission of genetic information depend on the correct action of different DNA damage response (DDR) pathways. Inefficient DNA damage repair, due to failure in recognition and repair of the damage, can result in the accumulation of genetic alterations and culminate in a cancer-prone phenotype. On the other hand, the effects of DNA damage signaling deregulation can have implications in cancer therapy resulting in hypersensitive or resistant tumor cells to therapeutic agents [2]. For instance, an inefficient activity of genes involved in DDR, such as the *RAD52* gene, can result in defective repair of DNA double-strand breaks (DSBs) by homologous recombination (HR). This defective mechanism increases genetic instability and predisposition to development of several cancer types. In addition, these DNA repair defects can be exploited therapeutically in order to improve cancer therapy targeting HR deficiency [3].

## 2. DNA Damage Signaling Pathways

Per day, various DNA-damaging agents can attack the cells and, consequently, originate a wide range of damages including single base lesions, DNA adducts, DNA crosslinks, single-strand breaks (SSBs), and double-strand breaks (DSBs). In order to ensure genomic integrity maintenance and to promote survival, cells present an intricate network of signaling pathways whose function is to counteract these damages, termed DNA damage response (DDR) [4]. However, if the DDR process is inefficient or nonfunctional, accumulation of DNA damage may result in genetic mutations and aberrant chromosomal segregations that can increase genomic instability, contributing to a higher risk of cancer development [4,5].

**Figure 1:**
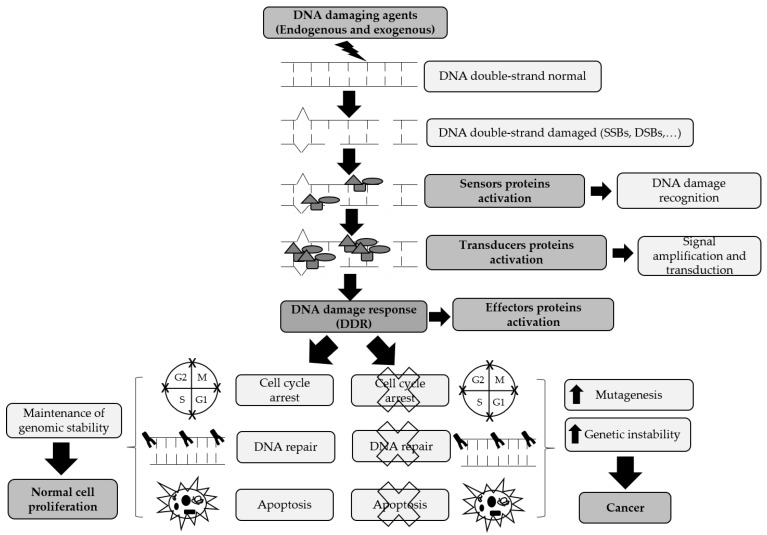
Organization and functional consequences of the DNA damage response (DDR). In DDR, different proteins act together to recognize the DNA damage (sensors), amplify and translate the DNA damage signal (transducers) and, consequently, stimulate an appropriate response (effectors). Several intrinsic mechanisms, including cell cycle checkpoints, DNA repair pathways, and apoptosis are activated to secure genomic stability maintenance and normal cell proliferation. However, when these mechanisms fail, DNA replication errors and aberrant chromosomal instability take place, culminating in increased mutagenesis and genomic instability and ultimately the promotion of cancer development.

DDR regulates repair process by the activation of several signaling networks: (1) Initial detection of the damage resulting in induction of cell cycle checkpoints; (2) DNA repair pathways activation, and (3) stimulation of cellular death by activation of programmed cell death pathway (apoptosis) [6]. One of the DDR outcomes can be cell survival, in which the correct DNA repair occurs, and the cell proceeds a normal replication. On the other hand, if inappropriate error repair occurs, it can either cause the cell to activate apoptosis as a response to the presence of very harmful damages or it can lead to the initiation and development of carcinogenesis (Figure 1) [7].

In DDR, the first step is cell cycle checkpoints activation in the different cell cycle phases due to incomplete DNA replication caused by the presence of DNA damage. These checkpoints can occur in transition G1/S and G2/M phases and S phase in order to block the cell cycle progression, allowing the recognition and suitable repair of the damage. Therefore, this prevents the replication of the damaged DNA and its transmission to the next generation cells [8,9]. Depending on the type of the DNA damage, cells will select different DNA repair mechanisms which are specific for each damage type. These repair mechanisms include nucleotide excision repair (NER), base excision repair (NER), mismatch repair (MMR), non-homologous end joining (NHEJ), and homologous recombination (HR) [10]. Usually, in the presence of an optimal DNA repair, cells can recover from the damage and continue normal cellular growth. However, when the genotoxic stress exceeds the repair capacity or the damage is irreparable, additional signaling pathways may lead to cell death by apoptosis to prevent the transmission of potentially mutagenic genetic alterations [8]. Apoptotic cell death is an energy-dependent process of cell suicide, in which, the content of the cell degrades without disrupting the outer cell membrane or promoting an inflammatory response [11].

Considering that DDR involves the action of multiple proteins responsible for recognition and signaling of DNA damages and consequent repair, a correct coordination of all activated cellular pathways is needed. In this sense, several classes of proteins have been extensively identified, including damage sensors, transducers, mediators, and effectors. Sensors are normally chromatid-bound proteins that have the function of DNA damage recognition and transducers recruitment. Afterwards, through post-translational modifications, such as phosphorylation, glycosylation, and ubiquitylation, transducer proteins are capable of intensifying damage response signals. Finally, by mediators’ action the transducers promote the recognition of effector proteins in order to activate the most appropriate DDR-pathway [12].

Nevertheless, the DDR can be seen as a cellular process with contradictory functions concerning carcinogenesis promotion and cancer therapy efficacy. Thus, if on one hand, DNA repair deficiency can promote damage accumulation and consequently genomic instability, which leads to a higher risk of cancer development. On the other hand, an efficient DDR, which correctly repairs damage, can negatively influence the objective of therapy by decreasing tumor cell death [13]. This promising strategy of DDR-pathways suppression resulting in an increase in conventional chemotherapeutics efficacy has become an attractive cancer therapeutic approach. Therefore, a deeper understanding of DNA repair mechanisms may be an essential alternative to achieve a successful cancer therapy.

## 3. Homologous Recombination and DNA Repair

Given the broad spectrum of DNA damage types, DSBs are considered the most deleterious and fatal for DNA integrity. This damage type is essentially caused by cellular processes associated with normal cell metabolism, such as DNA replication and genetic recombination that occurs during meiosis. Furthermore, DSBs can also be originated by cell exposition to exogenous agents used in cancer treatment, including IR and therapeutic drugs, such as platinum agents [14]. Unpaired or incorrectly repaired DSBs can cause loss of genetic information, cell growth arrest, cell death, and carcinogenesis. Thus, for DSBs repair and restoration of genomic integrity, there are two primary repair mechanisms including NHEJ and HR, which function in different cell cycle phases, the latter being considered the most important because it is an error-free pathway [15]. HR is a highly conserved pathway that is generally activated only in the late S and G2 phases of the cell cycle, since HR needs an intact homologous sequence located on the sister chromatids as a repair template to ensure an accurate repair [16].

**Figure 2:**
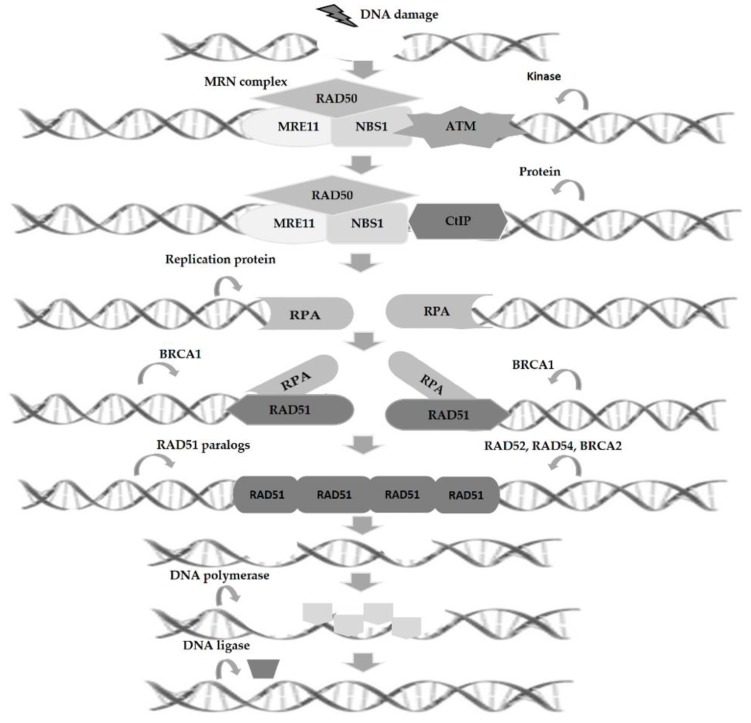
Double-strand breaks (DSBs) repair by homologous recombination. The homologous recombination (HR) pathway involves several steps: (1) Recognition and nucleolytic excision of DSB by the *MRE11/RAD50/NBS1* (MRN) complex together with ataxia-telangiectasia mutated (ATM) kinase activation; (2) 5′-3′ resection of the DNA ends by the combined action of MRN complex and CtIP protein, which results in formation of single-stranded DNA (ssDNA) overhangs on both break ends; (3) coverage and stabilization of the ssDNA overhangs by replication protein A (RPA) action; (4) displacement of RPA from ssDNA ends by *BRCA2* action inducing *RAD51* binding to ssDNA; (5) formation of the *RAD51*-ssDNA filament by *RAD51* paralogs, *RAD52, RAD54*, and *BRCA2*; (6) strand invasion promoted by *RAD51*-ssDNA filament to find an undamaged DNA template; (7) extension of the damaged 3′-end by DNA polymerase, and (8) strands annealing by DNA ligase resulting in DNA repair.

The first step in the HR repair process involves the recognition and nucleolytic excision of the DSB by the *MRE11/RAD50/NBS1* (MRN) complex, following the recruitment and activation of one of the damage sensor protein, such as ataxia-telangiectasia mutated (ATM) kinase. Then, the combined action of MRN complex and CtIP protein allows the 5′-3′ resection of the DNA ends, resulting in the creation of ssDNA protrusions on both ends of the break. Afterwards, replication protein A (RPA), a heterotrimeric complex (RPA1, RPA2, RPA3), is activated covering and stabilizing the ssDNA protrusions. In next step, *BRCA2* action induces the displacement of RPA from ssDNA ends and recombinase activity stimulation of RAD51 protein promoting their ssDNA binding. After that, through the joint action of *RAD51* paralogs (*RAD51B, RAD51C, RAD51D, XRCC2*, and *XRCC3*), *RAD52, RAD54*, and *BRCA2* occurs the formation of the *RAD51*-ssDNA filament. This filament promotes the strand invasion process with the homologous strand, in order to find an undamaged DNA template, leading to displacement of one strand as D-loop. Following D-loop formation, the affected 3′-end is extended by DNA synthesis through the primary break site in order to re-establish the missing sequence at the break point (Figure 2). Finally, DNA repair can occur by three different mechanisms, such as break-induced replication (BIR), synthesis-dependent strand annealing (SDSA), and double Holliday junctions (dHJ) [17]. BIR occurs through the invasion of one damaged DNA end into an intact homologous DNA strand. Subsequently, DNA polymerase initiates DNA synthesis which normally continues until the end of the homologous DNA sequence. This process is activated to repair one-ended breaks that can occur during the replication fork collapse and telomeric ends deterioration [18]. In SDSA, the strand responsible for invasion is extended through DNA synthesis, followed by D-loop inversion, promoting recently synthesized end liberation for annealing with the resected end in the 5′-3′ direction. This process is mediated by *RAD52* and promoted by homologue sequences annealing, culminating in the reconnection of the two broken ends. Finally, in recombination mediated by dHJ, the second DNA break invasion occurs throughout the D-loop. Next, DNA synthesis is promoted and followed by the binding of the two invading DNA ends which culminate in dHj resolution in a crossover or non-crossover way [19,20,21].

## 4. *RAD52* Functions and Its Implications in Carcinogenesis

### 4.1. RAD52 Proprieties and Functions

Radiation sensitive 52, also known as *RAD52*, was firstly identified in *S. cerevisiae* and is still a poor-characterized HR gene. The *RAD52* gene is localized in the genomic region 12p13.33 covering 37.6 kb of chromosome 12 and encoding a protein with 418 amino acids. This protein plays a role in DNA strand exchange and mediates the DNA-DNA interaction necessary for complementary DNA strands annealing during HR in mammalian cells [22]. Regarding the structure, RAD52 protein has a ring shape, containing several subunits. Furthermore, RAD52 is formed by two domains that divide the protein into two equivalent size parts, the N-terminal domain (NTD) and C-terminal domain (CTD) [23,24]. The NTD is a well-conserved region and is responsible for ssDNA binding and annealing actions of RAD52, while the CTD is poorly evolutionary conserved and is responsible for mediating interactions between *RAD51* and RPA in HR (Table 1) [25,26,27].

Recent studies demonstrated that *RAD52* has an important role in genomic stability maintenance and cancer suppression in mammalian cells, while several HR proteins including *BRCA1, BRCA2, PALB2*, and *RAD51* paralogs (*RAD51B*, *C*, *D*, and *XRCC 2* and *3*) present an inactivated and depleted activity [28,29,30]. When *BRCA2* and *PALB2* genes are depleted, defective activity of the human *RAD52* can be synthetically lethal [28]. Therefore, in human cells with defective *BRAC2* and *PALB2* genes, *RAD52* suppression can lead to an increased damage-associated genomic instability due to reduced HR activity, as a consequence, it promotes lower cell survival [31]. Furthermore, several evidences suggest that in mammalian cells, HR pathway presents a complex assembling and that, in the absence of the *BRACs* or other HR proteins, RAD52 protein can assume a back-up function to ensure damage repair. This *RAD52* role is verified in *S. cerevisiae*, given that, unlike many other eukaryotes, their genome does not encode *BRCA1/2* homologs. These findings reinforce the hypothesis that *RAD52* and *BRCA2* seem to function by similar pathways. Moreover, when the *BRCA2* is unavailable, *RAD52*′s action is a crucial alternative way to replication-induced damage repair by HR [32,33].

Despite extensive research on *RAD52*, the exact function remains to be elucidated. However, some studies demonstrate, the double function of *RAD52* as an annealing protein, belonging to a large single-strand annealing protein (SSAP) family and as a mediator of RAD51-dependent recombination [34,35]. After strand invasion and DNA synthesis, *RAD52* seems to play a postsynaptic role, one remains bonded to DNA, while *RAD51* disconnects from the intermediate recombination process [36]. In an attempt to perform strand-annealing reactions and, consequently, complete the interactions required for DBSs repair, *RAD52* binds to the moved strand of the D-loop and to ssDNA on the second end of DBSs. On the other hand, RAD52 protein has the exclusive role of promoting the annealing of ssDNA precoated with RPA. Thus, together with RPA, the binding and annealing activities of RAD52 are essential to promote the postsynaptic function of RAD52 in HR, in which an extended D-loop is available to be annealed to the second end of DSB [37]. In humans, RAD52 protein seems to associate preferentially to ssDNA, presenting a region of bound DNA with a stable sensitivity to hydroxyl radicals due to covering of ssDNA on the surface of RAD52 ring structure [38]. This packaging model is supported by a three-dimensional structure of the N-terminal domain of RAD52 [39].

### 4.2. RAD52 Expression and Regulation

Some studies have been reporting alterations in the expression patterns of *RAD52* gene after exposition of cancer cells to DNA-damaging agents. A study conducted by Fan et al. using malignant prostate cell lines irradiated or treated with mitomycin C demonstrated that mRNA and protein levels of *RAD52* gene were overexpressed in comparison to normal cell lines. However, chromosomal alterations assays showed that prostate cancer cells had a defective DNA damage repair despite *RAD52* elevated expression. This discordance between expression and activity of *RAD52* gene suggests that altered DNA repair mechanism promotes prostate tumor progression. This is probably due to the loss of HR activity control in malignant cells which is effective at the transcriptional level and may be insignificant to the altered function of transcription factors in cancer cells [40]. Ghosh et al. evaluated the impact of the RAD52 activation in human lung adenocarcinoma A549 cells when submitted to fractionated irradiation. The results showed that *RAD52* overexpression was considered one of the main factors responsible for the increase of the radioresistance in A459 cells. This observation demonstrates the functional importance of *RAD52* activity in DBS repair and its importance as an essential modulator factor of radioresistance [41]. On the other hand, another study showed that human hepatoblastoma (HepG2) cells treated with Etoposide or Methylmethanesulphonate (MMS) presented a significant increase in RAD52 expression, consistent with the amount of induced damage. These agents are considered chemical clastogens, which cause chromosome damage and induce DNA DSBs [42].

In response to DNA damage, *RAD52* promotes the formation of nuclear foci, which seem to correspond to DNA repair sites. This action of *RAD52* occurs under cell cycle control, where *RAD52* activity increases gradually when cells enter phase S, reaching a peak in the S phase and disappears at the beginning of G2 phase. Furthermore, *RAD52* can also undergo posttranslational modifications such as phosphorylation and sumoylation. Together, all these processes seem to regulate the timing of *RAD52* recruitment, its stability, and function [43,44].

During the S and G2/M phases of cell cycle, RAD52 recruitment is dependent on RPA, however in the G1 phase the RPA bound to ssDNA is not sufficient for its recruitment [45]. Additionally, CDK1-cyclin B kinase activity is essential for the recruitment of *RAD52*, which may act directly on *RAD52* or phosphorylate an upstream factor like RPA [46].

One of the main posttranslational modifications involved in the cellular response to DNA damage includes *RAD52* phosphorylation by c-Abl kinase at tyrosine 104. Tyr-104 residue is located in the N-terminal domain of RAD52 which is responsible for enhancing *RAD52* activity and stimulation of *RAD52* foci formation. Thus, this phosphorylation enhances *RAD52* ssDNA annealing activity by attenuating dsDNA binding [47,48]. Another modification of *RAD52* activity occurs by posttranslational addition of small ubiquitin-like modifier (SUMO) protein, which has a crucial role in mitotic and meiotic recombination. *MRE11-Rad50-Xrs2* (MRX) complex is induced by DNA damage resulting in *RAD52* sumoylation in the presence of DSBs both in meiotic and mitotic cells [49,50]. Moreover, *PTEN*, an important tumor suppressor, seems to physically interact with *RAD52* in response to DNA damage and is involved in the regulation of *RAD52* sumolyation in the nucleus [51].

Specifically, information concerning the transcriptional regulation of *RAD52* gene is still scarce. However, a study developed by Galanos et al. demonstrated that *RAD52* is activated transcriptionally in an E2F1 transcription factor-dependent way, rather than post-translationally as it is usual for DNA repair factor activation. In addition, these authors performed a bioinformatics analysis of *RAD52* promoter and observed that the promoter sequence comprised binding sites for several transcriptional factors, including E2F1 factor [52]. Thus, additional studies about the transcriptional regulation of *RAD52* gene are needed in order to identify new transcription factors associated with the modulation of its expression.

### 4.3. RAD52 in Carcinogenesis

Although *RAD52* has a very similar role to tumor suppressor *BRCA2* by repairing DNA damage in an attempt to ensure cell homeostasis and maintain cell viability, *RAD52* expression has only recently been related to different carcinogenesis processes. One of the first associations established between RAD52 and carcinogenesis was due to oxidative DNA damage and genomic instability in hematopoietic carcinogenesis [53]. Another study evaluated whether DNA repair pathways with genetic defects boosted the development of liver cancer in TGFalpha/c-myc mice. In this work, it was observed that, in comparison with wildtype controls, 10-week-old TGFalpha/c-myc and c-myc transgenic livers cells presented an upregulation of *RAD52* expression. Thus, this evidence was considered one of the first to propose that DDR-associated factors may directly promote an increased cancer risk. Later, Barlow group developed a study that established that murine *RAD52* gene expression is associated with Ataxia Telangiectasia (A-T). Therefore, tumor formation can occur with aberrant chromosome abnormalities due to loss of ATM kinase activity and uncontrolled HR, promoting an increased risk of cancer caused by A-T [54]. Another study developed by this group, showed, using an in vivo ATM−/− mouse model, that *RAD52* knockout seems to confer a longer latency period in T-cell lymphoma development, as well as an increase lifespan and decrease of tumor incidence when compared with the *RAD52* wildtype model [55]. This new evidence associated with the capacity to promote carcinogenesis and favor survival through *RAD52* inactivation in a tumorigenic environment was speculated to be a consequence of reduction in disproportionate intrachromosomal recombination found in the ATM absence.

As already mentioned, DNA repair activity is frequently altered in tumor cells leading to the high DNA damage levels [56]. One example of this DDR deregulation can be found in leukemia carcinogenesis mediated by *BCR-ABL1*, which presents a defective activity of *BRCA* gene. Therefore, depending on *RAD52* to repair the increased damage levels in the leukemia stem environment caused ROS’ presence [57]. This way, the use of a small peptide aptamer allows the target of one of the two DNA binding domains of *RAD52* and, consequently, inhibits its DNA binding capacity, promoting the accumulation of deleterious DBSs in leukemia cells. Furthermore, due to cells oncogenic dependence on DNA repair by *RAD52*, this inhibition of *RAD52′s* DNA binding can lead to the suppression of the clonogenic and proliferative potential of leukemia progenitor and stem cells.

Lieberman et al. used a TCGA database and showed a significant association between amplification of the genomic region where the *RAD52* gene is located (locus 12p13.33) and development of lung squamous cell carcinoma (LUSC). In addition, it was also demonstrated that, in LUSC patients, the somatic expression of *RAD51* gene was upregulated [58]. Similar results were obtained through the blockade of *RAD52* activity, which reduced cell growth and promoted senescence in mouse bronchial epithelial cells. In contrast, *RAD52* overexpression induced an increased cell proliferation rate. Furthermore, these authors revealed that in lung tumor cells of mouse, *RAD52* activity inactivation seems to alter the normal progress of cell cycle. This promotes an increased genomic instability owing to the excessive accumulation of DNA damage and, consequently, an increase in tumor cells’ death [58]. According to these genetic and functional evidences, *RAD52* can be considered a significant determinant of risk for lung cancer development.

In addition, several evidences have demonstrated that genetic variants in DNA repair genes, such as single nucleotide polymorphisms (SNPs), can influence cancer progression and treatment response. These genetic alterations are capable of altering the expression of a repair protein and, consequently, reduce or increase DNA repair activity of cells, affecting negatively or positively therapy outcome, respectively [59,60]. Concerning the *RAD52* gene, a study published by Shi et al. analyzed three *RAD52* SNPs with potential functional effect and evaluated, in an in vitro model, their association with platinum resistance and clinical outcome in cervical squamous cell carcinoma (CSCC) patients [61]. The results showed that two SNPs are associated with *RAD52* expression and nedaplatin or carboplatin resistance. Furthermore, these authors observed that patients with at least one variant allele have a significantly lower progression-free survival.

So, the correlation between genetic variants in DDR genes, such as *RAD52*, and protein expression could help to predict clinical outcome, treatment resistance, and monitor carcinogenesis in cancer patients.

## 5. *RAD52* as a Molecular Target for Cancer Therapy

The major problems in cancer treatment are toxic side effect due to lack of tumor cell specificity and poor efficiency of therapeutic agents owing to intrinsic or acquired resistance. Intrinsic resistance is characterized by resistance acquisition by tumor cells before primary treatment which conditions therapeutic drugs efficacy. Therefore, tumor eradication is not ensured even with precocious diagnosis and treatment. On the other hand, acquired resistance appears despite an initial positive therapy response [62].

Several DNA damage signaling pathways, such as damage recognition and repair mechanisms, seem to substantially influence the anticancer agent’s activity and, consequently, tumor cells elimination. In literature, it is described that DNA repair-defective tumor cells are associated with high genomic instability which can induce the acquisition of genetic alterations in specific genes, promoting treatment resistance. Mostly, this resistance is associated with drug transport and metabolism alterations that can lead to reduced drug effect. In addition, the presence of additional DNA modifications in DNA damage signaling pathways may also promote resistance to therapy in tumor cells with defective DDR activity [63,64].

In oncology, based on these assumptions, the synthetic lethality concept has been extensively studied, mainly to target HR pathway in tumor cells with a previously depleted repair activity [65]. This concept assumes that, in the presence of a defective repair pathway, the inactivation of an additional pathway can result in cell death by DNA damage accumulation caused by the inactivation of two complementary repair pathways. On the other hand, the depletion of a single pathway is not enough to cause cell death, given that, in the presence of a defective repair activity, the cell remains capable of tolerating damage and promoting cell survival. In clinical practice, this concept is frequently exploited, when tumor cells are deficient in one of the repair mechanisms owing to a genetic alteration and another mechanism is pharmacologically inactivated by the targeted cytotoxic agent. Therapeutically, this approach can be advantageous because in normal conditions the cancer cells death is induced, while the healthy cells are unaffected, as they have both pathways with a normal activity [66]. However, the introduction of this DDR-targeted therapeutic approach in clinical practice for treatment of cancers can be discouraged due at least two problems. First, cancer is a disease characterized by a quite heterogeneous biology. Secondly, the transition from preclinical studies into clinical trials of these potential new target or small-molecule-targeted therapy is quite challenging [7].

**Figure 3:**
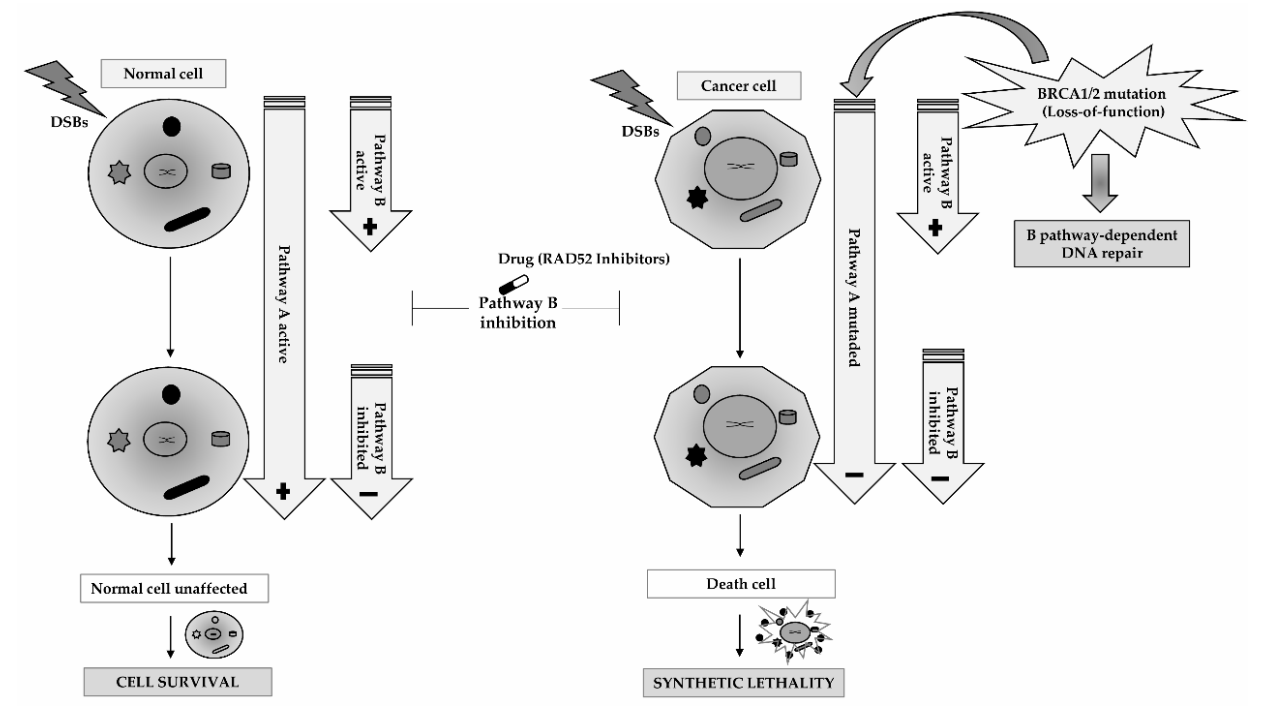
Synthetic lethality by targeting the *RAD52* gene. The resolution of the DSBs depends on two pathways (Pathways A and B) associated with homologous recombination repair. In *BRCA-*deficient cancer cells, the HR pathway activity is reduced due to the presence of mutations in *BRCAs* genes (Pathways A) leading to loss-of-function. An alternative pathway dependent on *RAD52* (Pathway B) needs to be activated, when the major repair pathway dependent on *BRCA* genes are inactive. However, if the *RAD52*-dependent pathway is simultaneously inhibited using inhibitors drugs of *RAD52* activity can induce cancer cells death. Therefore, the inhibition of *RAD52* is synthetically lethal with *BRCA*-deficiency. On the other hand, in normal cells the inhibition of *RAD52* activity (Pathway B) is compensated by normal function of *BRCAs* genes (Pathways A) and the cells are unaffected promoting cellular survival.

Initially, synthetic lethality concept development was encouraged to kill cancer cells with inactivating mutations in *BRCA1* and *BRAC2* by poly adenosine 5′-disphosphate ribose polymerase (PARP) inhibition [67]. Actually, there are preliminary evidence reporting the application of synthetic lethality dependent of *RAD52* gene in cells with mutations in *BRCA1* and *BRCA2* genes, as well as in cells with suppressed *BRCA1-RAD51* pathway due to oncogenes activation or epigenetic modifications associated to malignancy of phenotypes [57]. To target *RAD52* in *BRCA*-deficient tumors, the inactivation of *RAD52* in the clinical setting can be modulated through some approaches. The first approach would be to target *RAD52* directly, using aptamares which target the binding capacity of *RAD52* to DNA. However, until now, it has been difficult to develop pharmacologic approaches related to the *RAD52* gene because the enzymatic or kinase actions of *RAD52* remain unknown, hence necessitating more functional studies to determine *RAD52* activity. Another approach is based on molecular pharmacology advances, which can create or identify small molecule inhibitors targeting *RAD52* that break the oligomer ring structure or bind near DNA binding groove of *RAD52* to avoid access by the DNA substrate [29,68,69]. So, due to increased interest in developing new ways of targeting *RAD52* activity and expression, Huang et al. identified seventy potential inhibitor molecules capable of altering *RAD52* function and consequently inhibiting the ssDNA annealing capacity of *RAD52*. In practical context, it was possible to observe that in hereditary ovarian and breast cancers with a defective activity of *BRCA1*/2 genes and inactivation of *RAD52* function, by specific inhibitors, there was an induced suppression of tumor cells progression (Figure 3) [69].

In human cancer cell-lines with defective function of *BRCA1*, *PALB2* or *BRCA2*, *RAD52′s* depletion increases damage-associated chromosomal abnormalities, decreases clonal viability, and also reduces the HR activity [28,30]. Thus, it appears to exist a relationship between *RAD52* and *BRCA1, PLAB2*, and *BRCA2* covering the synthetic lethality concept. *PALB2* activation is necessary for the recruitment of *BRCA2* to foci, however *BRCA2* activity and consecutive HR functioning mediated by *RAD51* can be altered by suppression of the *BRCA1-PLAB2* physical interaction [70,71]. Therefore, when *BRCA*-pathway is depleted, *RAD52* activity can act as an alternative mediator, replacing the role of *BRCA1-PALB2-BRCA2* pathway and promoting tumor cells growth. However, in *BRCA1*, *PALB2* or *BRCA2* mutant cells, the decrease on *RAD52* activity can lead to cell death. Therapeutically, these data allow to reinforce the fact that any cancer type, with *BRCA1-PALB2-BRCA2* pathway suppressed, may be targeted by inactivation of *RAD52*. Despite the development of other therapeutic approaches that take advantage of the synthetic lethality concept in cancers carrying *BRCAs* genes alterations, such as poly-(ADP-ribose) polymerase (PARP) inhibition, this strategy using *RAD52* inhibitors to target depleted *BRCA*-pathway is different [72,73].

Some studies have demonstrated that personalized synthetic lethality induced by targeting *RAD52* is achieved in *BRCA*-deficient carcinomas and leukemias, while normal cells and tissues remain unaffected [30,57]. In concordance with these observations, another study performed by Sullivan-Reed et al. showed that the simultaneous targeting of *PARP1* and *RAD52* genes exerted a synergistic action by trigging a dual synthetic lethality in *BRCA*-deficient tumor cells. Interestingly, this synergic treatment seems to not affect normal cells and tissues and tumor cells with competent activity of *BRCA* genes [74]. Moreover, a common event of *PARP* inhibitors treatment is the development of resistance. Therefore, the dual synthetic lethality originated by the combined inhibition of *PARP* and *RAD52* genes is seen as an advantageous therapeutic strategy to overcome this issue. This way, the *BRCA*-deficient malignant cells can be more easily eliminated and, consequently, limiting or preventing the appearance of prior resistant clones or induced by cytotoxic drug. Finally, this study also showed that the therapeutic outcome may be improved by RAD52 inactivation of *BRCA* genes-defective cancers and treated with agents that inhibit *PARP*, while inducing a minimal toxicity to normal cells.

## 6. Conclusions

DNA damage signaling pathways, including DNA repair machinery, are essential to genomic stability maintenance, growth suppression of cells with genetic defects and, consequently, in carcinogenesis prevention. On the other hand, the treatment outcome is highly influenced by all cellular processes involved in DDR. Therefore, a therapeutic strategy that targets tumor-specific DNA repair pathways through *RAD52* inactivation may be a promising approach to improve therapy efficacy since it can result in an increased tumor cells sensitization to cell death and a decreased toxicity to normal cells.

New approaches of *RAD52* inhibition would potentially provide a complementary strategy for targeting *BRCA*-deficient cancers in addition to *PARP* inhibitors.

## Figures and Tables

**Table 1:** Characteristics of the gene *RAD52*.

Characteristic Type	Description
Name	Radiation sensitive 52
Symbol	*RAD52*
Molecular weight	46169 Da
Size	418 amino acids
Structure	Heptameric rings. Two domains (NTD and CTD)
Map locus	Chromosome 12p12.2-13
Coding protein	DNA repair protein *RAD52* homolog
Function	DBSs repair by promoting the annealing of complementary single-stranded DNA and by *RAD51* recombinase stimulation
Primary localization	Nucleus
Protein interactions	ABL1, RPA2, RAD51
Described polymorphisms	rs1060499, rs11571493, rs11571496, rs11571497, rs139916251

## References

[1] Goldstein M., Kastan M.B. (2015). The DNA Damage Response: Implications for Tumor Responses to Radiation and Chemotherapy. Annu. Rev. Med..

[2] Bouwman P., Jonkers J. (2012). The effects of deregulated DNA damage signalling on cancer chemotherapy response and resistance. Nat. Rev. Cancer.

[3] Lieberman R., You M. (2017). Corrupting the DNA damage response: A critical role for Rad52 in tumor cell survival. Aging.

[4] Hoeijmakers J.H. (2009). DNA Damage, Aging, and Cancer. New Engl. J. Med..

[5] Shen Z. (2011). Genomic instability and cancer: An introduction. J. Mol. Cell Boil..

[6] Ciccia A., Elledge S.J. (2010). The DNA Damage Response: Making it safe to play with knives. Mol. Cell.

[7] Kinsella T.J. (2009). Understanding DNA Damage Response and DNA Repair Pathways: Applications to More Targeted Cancer Therapeutics. Semin. Oncol..

[8] Reinhardt H.C., Yaffe M.B. (2009). Kinases that control the cell cycle in response to DNA damage: Chk1, Chk2, and MK2. Curr. Opin. Cell Boil..

[9] Kastan M.B., Bartek J. (2004). Cell-cycle checkpoints and cancer. Nature.

[10] Jeggo P.A. (1998). DNA breakage and repair. Adv. Genet..

[11] Wang X. (2001). The expanding role of mitochondria in apoptosis. Genes Dev..

[12] Harper J.W., Elledge S.J. (2007). The DNA Damage Response: Ten Years After. Mol. Cell.

[13] Tian H., Gao Z., Li H., Zhang B., Wang G., Zhang Q., Pei D., Zheng J. (2015). DNA damage response—A double-edged sword in cancer prevention and cancer therapy. Cancer Lett..

[14] Khanna K.K., Jackson S.P. (2001). DNA double-strand breaks: Signaling, repair and the cancer connection. Nat. Genet..

[15] Shrivastav M., De Haro L.P., Nickoloff J.A. (2008). Regulation of DNA double-strand break repair pathway choice. Cell Res..

[16] Moynahan M.E., Jasin M. (2010). Mitotic homologous recombination maintains genomic stability and suppresses tumorigenesis. Nat. Rev. Mol. Cell Boil..

[17] Heyer W.-D., Ehmsen K.T., Liu J. (2010). Regulation of homologous recombination in eukaryotes. Annu. Rev. Genet..

[18] Llorente B., Smith C.E., Symington L.S. (2008). Break-induced replication: What is it and what is it for?. Cell Cycle.

[19] San Filippo J., Sung P., Klein H. (2008). Mechanism of eukaryotic homologous recombination. Annu. Rev. Biochem..

[20] Sung P., Klein H. (2006). Mechanism of homologous recombination: Mediators and helicases take on regulatory functions. Nat. Rev. Mol. Cell Biol..

[21] Hartlerode A.J., Scully R. (2009). Mechanisms of double-strand break repair in somatic mammalian cells. Biochem. J..

[22] Mortensen U.H., Bendixen C., Sunjevaric I., Rothstein R. (1996). DNA strand annealing is promoted by the yeast Rad52 protein. Proc. Natl. Acad. Sci. USA.

[23] Lloyd J.A., McGrew D.A., Knight K.L. (2005). Identification of Residues Important for DNA Binding in the Full-length Human Rad52 Protein. J. Mol. Boil..

[24] Stasiak A.Z., Larquet E., Stasiak A., Müller S., Engel A., Van Dyck E., West S.C., Egelman E.H. (2000). The human Rad52 protein exists as a heptameric ring. Curr. Boil..

[25] Asleson E.N., Okagaki R.J., Livingston D.M. (1999). A core activity associated with the N terminus of the yeast RAD52 protein is revealed by RAD51 overexpression suppression of C-terminal rad52 truncation alleles. Genetics.

[26] Krejci L., Song B., Bussen W., Mortensen U.H., Rothstein R., Sung P. (2002). Interaction with Rad51 Is Indispensable for Recombination Mediator Function of Rad52*. J. Boil. Chem..

[27] Shen Z., Denison K., Lobb R., Gatewood J.M., Chen D.J. (1995). The human and mouse homologs of the yeast RAD52 gene: cDNA cloning, sequence analysis, assignment to human chromosome 12p12.2–p13, and mRNA expression in mouse tissues. Genomics.

[28] Lok B.H., Carley A.C., Tchang B., Powell S.N. (2013). RAD52 inactivation is synthetically lethal with deficiencies in BRCA1 and PALB2 in addition to BRCA2 through RAD51-mediated homologous recombination. Oncogene.

[29] Lok B.H., Powell S.N. (2012). Molecular pathways: Understanding the role of Rad52 in homologous recombination for therapeutic advancement. Clin. Cancer Res..

[30] Feng Z., Scott S.P., Bussen W., Sharma G.G., Guo G., Pandita T.K. (2011). Rad52 inactivation is synthetically lethal with BRCA2 deficiency. Proc. Natl. Acad. Sci. USA.

[31] Shi J., Chatterjee N., Rotunno M., Wang Y., Pesatori A.C., Consonni D. (2012). Inherited variation at chromosome 12p13.33, including RAD52, influences the risk of squamous cell lung carcinoma. Cancer Discov..

[32] Iyer L.M., Koonin E.V., Aravind L. (2002). Classification and evolutionary history of the single-strand annealing proteins, RecT, Redbeta, ERF and RAD52. BMC Genom..

[33] Sugiyama T., Kowalczykowski S.C. (2002). Rad52 Protein Associates with Replication Protein A (RPA)-Single-stranded DNA to Accelerate Rad51-mediated Displacement of RPA and Presynaptic Complex Formation. J. Boil. Chem..

[34] Symington L.S. (2002). Role of RAD52 Epistasis Group Genes in Homologous Recombination and Double-Strand Break Repair. Microbiol. Mol. Boil. Rev..

[35] McIlwraith M.J., West S.C. (2008). DNA Repair Synthesis Facilitates RAD52-Mediated Second-End Capture during DSB Repair. Mol. Cell.

[36] Miyazaki T., Bressan D.A., Shinohara M., Haber J.E., Shinohara A. (2004). In vivo assembly and disassembly of Rad51 and Rad52 complexes during double-strand break repair. EMBO J..

[37] Wu Y., Sugiyama T., Kowalczykowski S.C. (2006). DNA Annealing Mediated by Rad52 and Rad59 Proteins. J. Boil. Chem..

[38] Parsons C.A., Baumann P., Van Dyck E., West S.C. (2000). Precise binding of single-stranded DNA termini by human RAD52 protein. EMBO J..

[39] Singleton M.R., Wentzell L.M., Liu Y., West S.C., Wigley D.B. (2002). Structure of the single-strand annealing domain of human RAD52 protein. Proc. Natl. Acad. Sci. USA.

[40] Fan R., Kumaravel T.S., Jalali F., Marrano P., Squire J.A., Bristow R.G. (2004). Defective DNA Strand Break Repair after DNA Damage in Prostate Cancer Cells: Implications for Genetic Instability and Prostate Cancer Progression. Cancer Res..

[41] Ghosh S., Krishna M. (2012). Role of Rad52 in fractionated irradiation induced signaling in A549 lung adenocarcinoma cells. Mutat. Res. Mol. Mech. Mutagen..

[42] Smith C.C., Aylott M.C., Fisher K.J., Lynch A.M., Gooderham N.J. (2006). DNA damage responses after exposure to DNA-based products. J. Gene Med..

[43] Liu Y., Li M.-J., Lee E.Y.-H., Maizels N. (1999). Localization and dynamic relocalization of mammalian Rad52 during the cell cycle and in response to DNA damage. Curr. Boil..

[44] Barlow J.H., Rothstein R. (2010). Timing is everything: Cell cycle control of Rad52. Cell Div..

[45] Barlow J.H., Lisby M., Rothstein R. (2008). Differential regulation of the cellular response to DNA double-strand breaks in G1. Mol. Cell.

[46] Hanamshet K., Mazina O.M., Mazin A.V. (2016). Reappearance from Obscurity: Mammalian Rad52 in Homologous Recombination. Genes.

[47] Kitao H., Yuan Z.-M. (2002). Regulation of Ionizing Radiation-induced Rad52 Nuclear Foci Formation by c-Abl-mediated Phosphorylation. J. Boil. Chem..

[48] Honda M., Okuno Y., Yoo J., Ha T., Spies M. (2011). Tyrosine phosphorylation enhances RAD52-mediated annealing by modulating its DNA binding. EMBO J..

[49] Altmannova V., Eckert-Boulet N., Arneric M., Kolesar P., Chaloupkova R., Damborsky J., Sung P., Zhao X., Lisby M., Krejci L. (2010). Rad52 SUMOylation affects the efficiency of the DNA repair. Nucleic Acids Res..

[50] Ohuchi T., Seki M., Branzei D., Maeda D., Ui A., Ogiwara H., Tada S., Enomoto T. (2008). Rad52 sumoylation and its involvement in the efficient induction of homologous recombination. DNA Repair.

[51] Choi B.H., Chen Y., Dai W. (2013). Chromatin PTEN is involved in DNA damage response partly through regulating Rad52 sumoylation. Function of a membrane-embedded domain evolutionarily multiplied in the GPI lipid anchor pathway proteins PIG-B, PIG-M, PIG-U, PIG-W, PIG-V, and PIG-Z. Cell Cycle.

[52] Galanos P., Pappas G., Polyzos A., Kotsinas A., Svolaki I., Giakoumakis N.N., Glytsou C., Pateras I.S., Swain U., Souliotis V.L. (2018). Mutational signatures reveal the role of RAD52 in p53-independent p21-driven genomic instability. Genome Boil..

[53] Hironaka K., Factor V.M., Calvisi D.F., Conner E.A., Thorgeirsson S.S. (2003). Dysregulation of DNA repair pathways in a transforming growth factor alpha/c-myc transgenic mouse model of accelerated hepatocarcinogenesis. Lab. Investig..

[54] Barlow C., Hirotsune S., Paylor R., Liyanage M., Eckhaus M., Collins F., Shiloh Y., Crawley J.N., Ried T., Tagle D. (1996). Atm-Deficient Mice: A Paradigm of Ataxia Telangiectasia. Cell.

[55] Treuner K., Helton R., Barlow C. (2004). Loss of Rad52 partially rescues tumorigenesis and T-cell maturation in Atm-deficient mice. Oncogene.

[56] Alcalay M., Meani N., Gelmetti V., Fantozzi A., Fagioli M., Orleth A., Riganelli D., Sebastiani C., Cappelli E., Casciari C. (2003). Acute myeloid leukemia fusion proteins deregulate genes involved in stem cell maintenance and DNA repair. J. Clin. Investig..

[57] Cramer-Morales K., Nieborowska-Skorska M., Scheibner K., Padget M., Irvine D.A., Sliwinski T., Haas K., Lee J., Geng H., Roy D. (2013). Personalized synthetic lethality induced by targeting RAD52 in leukemias identified by gene mutation and expression profile. Blood.

[58] Lieberman R., Xiong D., James M., Han Y., Amos C.I., Wang L. (2016). Functional characterization of RAD52 as a lung cancer susceptibility gene in the 12p13.33 locus. Mol. Carcinog..

[59] Gossage L., Madhusudan S. (2007). Cancer pharmacogenomics: Role of DNA repair genetic polymorphisms in individualizing cancer therapy. Mol. Diagn. Ther..

[60] Zhang L., Ma W., Li Y., Wu J., Shi G. (2014). Pharmacogenetics of DNA repair gene polymorphisms in non-small-cell lung carcinoma patients on platinum-based chemotherapy. Genet. Mol. Res..

[61] Shi T.-Y., Yang G., Tu X.-Y., Yang J.-M., Qian J., Wu X.-H., Zhou X.-Y., Cheng X., Wei Q. (2012). RAD52 Variants Predict Platinum Resistance and Prognosis of Cervical Cancer. PLoS ONE.

[62] Sun Y. (2016). Tumor microenvironment and cancer therapy resistance. Cancer Lett..

[63] Rebucci M., Michiels C. (2013). Molecular aspects of cancer cell resistance to chemotherapy. Biochem. Pharmacol..

[64] Lord C.J., Ashworth A. (2012). The DNA damage response and cancer therapy. Nature.

[65] Shaheen M., Allen C., Nickoloff J.A., Hromas R. (2011). Synthetic lethality: Exploiting the addiction of cancer to DNA repair. Blood.

[66] Bhattacharjee S., Nandi S. (2017). Synthetic lethality in DNA repair network: A novel avenue in targeted cancer therapy and combination therapeutics. IUBMB Life.

[67] Farmer H., McCabe N., Lord C.J., Tutt A.N.J., Johnson D.A., Richardson T.B., Santarosa M., Dillon K.J., Hickson I., Knights C. (2005). Targeting the DNA repair defect in BRCA mutant cells as a therapeutic strategy. Nature.

[68] Sullivan K., Cramer-Morales K., McElroy D.L., Ostrov D.A., Haas K., Childers W., Hromas R., Skorski T. (2016). Identification of a Small Molecule Inhibitor of RAD52 by Structure-Based Selection. PLoS ONE.

[69] Huang F., Goyal N., Sullivan K., Hanamshet K., Patel M., Mazina O.M., Wang C.X., An W.F., Spoonamore J., Metkar S. (2016). Targeting BRCA1- and BRCA2-deficient cells with RAD52 small molecule inhibitors. Nucleic Acids Res..

[70] Zhang F., Ma J., Wu J., Ye L., Cai H., Xia B., Yu X. (2009). PALB2 links BRCA1 and BRCA2 in the DNA-damage response. Curr. Boil..

[71] Sy S.M.H., Huen M.S.Y., Chen J. (2009). PALB2 is an integral component of the BRCA complex required for homologous recombination repair. Proc. Natl. Acad. Sci. USA.

[72] Bryant H.E., Schultz N., Thomas H.D., Parker K.M., Flower D., Lopez E., Kyle S., Meuth M., Curtin N.J., Helleday T. (2005). Specific killing of BRCA2-deficient tumours with inhibitors of poly(ADP-ribose) polymerase. Nature.

[73] Fong P.C., Boss D.S., Yap T.A., Tutt A., Wu P., Mergui-Roelvink M., Mortimer P., Swaisland H., Lau A., O’Connor M.J. (2009). Inhibition of Poly(ADP-Ribose) Polymerase in Tumors fromBRCAMutation Carriers. New Engl. J. Med..

[74] Sullivan-Reed K., Bolton-Gillespie E., Dasgupta Y., Langer S., Siciliano M., Nieborowska-Skorska M., Hanamshet K., Belyaeva E.A., Bernhardy A.J., Lee J. (2018). Simultaneous Targeting of PARP1 and RAD52 Triggers Dual Synthetic Lethality in BRCA-Deficient Tumor Cells. Cell Rep..

